# More Prosocial, More Ephemeral? Exploring the Formation of a Social Entrepreneur’s Exit Intention via Life Satisfaction

**DOI:** 10.3390/ijerph19126966

**Published:** 2022-06-07

**Authors:** Jianing Dong, Xiao Wang, Xuanwei Cao, David Higgins

**Affiliations:** 1 International Business School Suzhou, Xi’an Jiaotong-Liverpool University, Suzhou 215123, China; jianing.dong20@student.xjtlu.edu.cn (J.D.); xuanwei.cao@xjtlu.edu.cn (X.C.); 2 Management School, University of Liverpool, Liverpool L69 3BX, UK; dhiggins@liverpool.ac.uk

**Keywords:** social entrepreneur, exit intention, prosocial motivation, life satisfaction, gender

## Abstract

This study was designed to test if satisfaction with health and personal financial wellbeing mediates the relationship between prosocial motivations and exit intentions among social entrepreneurs. Using a sample of 317 social entrepreneurs, the partial least square structural equation modeling (PLS-SEM) revealed that prosocial motivation decreased the financial satisfaction of entrepreneurs, which increased their exit intentions. However, health satisfaction did not have a mediating effect on the relationship between prosocial motivation and exit intention. Moreover, adopting the multi-group analysis (MGA) technique, we found that the negative impact of prosocial motivation on financial satisfaction was stronger for males than for females, suggesting male entrepreneurs were more likely to experience lower financial satisfaction caused by prosocial motivation than female entrepreneurs. There was no evidence that gender moderated the relationship between prosocial motivation and health satisfaction.

## 1. Introduction

Why do most entrepreneurs end up unsuccessful in their businesses? Despite a large number of studies on entrepreneurial opportunities, entrepreneurial characteristics, and determinants of new emergent ventures in specific social and global contexts [1], scholars have argued that it is also crucial to explore the determinants of entrepreneurs’ intentions to leave or terminate their ventures [2]. From another perspective, understanding what determines an entrepreneur’s exit intention is important to understanding the nature of entrepreneurial success [3]. Moreover, compared to studies conducted on conventional or regular entrepreneurs, much less is known about the exit intentions of social entrepreneurs [4], and even less is known about the psychological antecedents of the exit intentions [2] or about the emotional processes that lead to the exit intentions [5].

Prosocial motivation, the core personality trait of social entrepreneurs, is a psychological antecedent that has been the subject of considerable research [6,7]. It differentiates from conventional or regular entrepreneurs [8] and drives entrepreneurs to have concern for others/the endeavor to help. However, extant studies on the relationship between prosocial motivation and entrepreneurs’ exit intentions have reported contentious findings. Some investigators have suggested that social entrepreneurs develop an “attachment to their organizations” as a result of the process of helping others in their work, which, in turn, may emotionally impede their intention to exit [9,10,11]. However, some studies indicated that prosocial motivation may hinder the venture development of a viable firm or lead to its failure [12,13], thereby increasing a sense of failure and arousing the intention to exit [14]. Consequently, there are substantial gaps in our understanding of the relationship between prosocial motivation and the exit intentions of social entrepreneurs [15]; more in-depth explorations are needed.

According to Bolino and Grant [7], prosocial motivation has been mostly investigated as a personality trait that may trigger behaviors that are both positive (organizational citizenship behaviors) and negative (less engagement in task performance). However, its capacity to predict exit intentions can be quite limited [16,17,18,19,20]; its direct effects on behavioral outcomes have been questioned [20,21,22] and there is a need to examine if a mediating mechanism links prosocial motivation and exit intentions [17].

According to Carree and Verheul [23] and Lindblom, Lindblom, and Wechtler [20], entrepreneurship is much more than a 9-to-5 job; it can often be regarded as a 24/7 fulfilling lifestyle that leads to a situation where satisfaction with one’s job and other life domains are intertwined [24]. Social entrepreneurs who devote themselves to more than regular entrepreneurial work likely spend much more time and energy helping others [7,25,26,27] than necessary [7,13,28]. Previous researchers have suggested that life satisfaction is a critical mediator between prosocial motivations and exit intentions among social entrepreneurs [20,29]. Thus, the central question raised by this study is: does life satisfaction mediate the relationship between prosocial motivation and exit intention?

A special report on social entrepreneurship [30] claims that social enterprises are more likely to be established by men, but longitudinally, the gender gap is not as significant as it was in the early stages. This implies that male entrepreneurs are more likely to quit social entrepreneurship than their female peers. However, research as to why more women entrepreneurs persist as social entrepreneurs is rare [31,32]. Based on the view of gender stereotypes [33,34], we explore the relevant effects of social entrepreneurs and gender.

This study contributes to the literature in three ways: First, responding to Tina, Foss, and Stefan [6], who suggested exploring the potential negative effects of prosocial motivation, especially regarding the entrepreneur’s intention to sustain, this study can help address the debate on the relationship between a social entrepreneur’s prosocial motivation and exit intention by examining if life satisfaction mediates this relationship [9,10,13]. Second, this study extends the discussion on gender differences in social entrepreneurship [35], especially how it moderates the effects of prosocial motivation on life satisfaction, facilitating the exit intentions of social entrepreneurs. Third, drawing on empirical data from a relatively large sample, we contribute to the scarce quantitative literature on social entrepreneurship [36,37].

## 2. Theoretical Background and Hypothesis Development

A hierarchical approach to personality assumes that personality traits (1) determine how we respond in various contexts, and (2) shape our behaviors [21,22]. A key assumption is that personality is hierarchically arranged [21,22]. At the top of the hierarchy are basic personality traits, which serve as the building blocks, shaping most of our behaviors [38]. At the bottom of the hierarchy are surface traits, which are more specific and have significant behavioral consequences. The basic personality traits, compared to the surface traits, are enduring dispositions that determine behaviors in a wider range of situations. Surface traits are context-specific and result in behaviors from interactions between basic traits and contextual elements [38,39,40]. Researchers, including Licata et al. [41], Brown et al. [42], and Prentice and King [43], argue that traits function hierarchically: basic personality traits serve at a deeper level, and provide a foundation for surface traits that function as mediators and relate more closely to individual behaviors.

Following the hierarchical approach to personality [21,22], prosocial motivation is regarded as a basic personality trait and it represents “a person’s ‘affective lens’ (remains constant over the time) on the world” [7,44,45], determining a person’s responses in various contexts [21,22]; in contrast, life satisfaction is viewed as a surface trait that connects prosocial motivation and exit intentions [20].

### 2.1. Prosocial Motivation and Exit Intention

Frequent heroic characterizations of social entrepreneurs have limited the foci of those who have fewer positive stories to tell [46,47,48,49]. Normally, social entrepreneurs and their ventures encounter a range of unique challenges [50], uncertainties, and problems [51], resulting in entrepreneurial exits [6]. Lindblom, Lindblom, and Wechtler [20] and Pollack et al. [52] defined exit intention as “an entrepreneur’s desire or goal, at some point in the future, to leave his or her venture.” According to Renko [13], entrepreneurs with strong prosocial motivations are less likely to succeed in sustaining their businesses, compared with entrepreneurs who are mainly motivated by financial goals. This is because social entrepreneurs are characterized by pursuing a dual mission of economic and social value creation [53], inducing conflicting and competing logic [54,55,56]. Largely, social entrepreneurs need to combine their prosocial motivations with regular practices regarding for-profit firms [57,58,59] and foster inconsistent goals, norms, and values that may lead to contradictory prescriptions for actions [60]. This can cause tension [60], resulting in stronger exit intentions [6,61]. Nevertheless, few studies have investigated such underlying mechanisms. Responding to the calls for systemically exploring the “dark side” of prosocial motivation [7], this research investigates how prosocial motivation affects a social entrepreneur’s intention to exit.

### 2.2. Life Satisfaction

As prior studies have suggested, there may be a key mediator between prosocial motivation and exit intention [7,13,17,28]. Since social entrepreneurship can consume one’s time and even impair one’s personal life [7,25,26,27], prosocial motivation may considerably undermine life satisfaction. Thus, social entrepreneurs may consider ceasing their work to regain their diminished life satisfaction, expediting their exit intentions [62,63]. However, empirical studies examining this relationship are rare [64].

Diener et al. [65] defined life satisfaction as a cognitive judgmental process through which an entrepreneur assesses his or her quality of life as a whole. For entrepreneurs, being satisfied with life indicates that they appreciate the progress they have made in achieving their life goals in both work and family domains [66,67]. The two-layer model suggested by Ferrer-I-Carbonell et al. [68] and Erdogan et al. [69] indicates that life satisfaction has two dimensions: financial satisfaction and health satisfaction.

### 2.3. Prosocial Motivation, Financial Satisfaction, and Exit Intention

Financial satisfaction can be defined as a cognitive evaluation of one’s present financial situation [70]. According to the Wharton Center, NYU Stern, and the Fuqua School, all social entrepreneurs do well (financially) by doing good (socially) [58], although it is a critical challenge [58,71,72,73]. Previous research based on a survey in the United Kingdom provided evidence that social entrepreneurs considered securing financial capital (to develop their businesses) as a major challenge [74]. Largely, adequate income and financial sustainability are buffers against the anxiety and psychological strains of running social businesses [50,75]. According to relevant studies, entrepreneurs who suffer from psychological strains due to financial difficulties will have lower levels of financial satisfaction, especially when financial difficulties are perceived as a signal of entrepreneurial failure [76].

Empirical research generally supports the relationship between financial satisfaction and exit intention among those who pursue prosocial careers. The motivation to pursue self-employment is often tied to economic concerns and the desire to create wealth [77,78]. However, for social entrepreneurs, the major pursuit is to achieve both financial and social goals [79]. Although economic outcomes are not regarded as the exclusive missions of social entrepreneurs [80], they may regret their initial decisions to start such a business when they do not succeed financially [80,81]. Largely, social entrepreneurs focus on outcomes [79], and their commitments to their prosocial ideas, businesses, and products are often intense [82,83]. However, empirical evidence has shown that lower levels of financial satisfaction decrease social entrepreneurs’ confidence in their own competence [84] because financial barriers can erode their commitments to their prosocial ideas, businesses, and products [80]. This negative emotion can be magnified, likely leading to intense regret and decreased intention to sustain their social ventures [81]. Therefore, we hypothesize:

**Hypothesis** **1a** **(H1a).** *Social entrepreneurs’ prosocial motivations have a negative effect on their financial satisfaction*.

**Hypothesis** **1b** **(H1b).** *Social entrepreneurs’ prosocial motivations have an indirect effect on their exit intentions via financial satisfaction*.

### 2.4. Prosocial Motivation, Health Satisfaction, and Exit Intention

Health satisfaction is a cognitive judgment about the quality of one’s overall mental and physical fitness [85,86]. Social entrepreneurs tend to have heavier workloads [32], encounter greater business risks, experience higher levels of job stress [87] and incur more psychosomatic ailments than other types of entrepreneurs [7]. Davis et al. [88], Ashton [89], and Kibler et al. [90] found that social entrepreneurs are passionate about their goals, but they are vulnerable to stress resulting from investing considerable time, effort, and cognitive resources needed to fulfill their many commitments on a daily basis. The passion for a prosocial business not only implies higher health costs [91,92] but can also induce anxiety when social entrepreneurs find their job responsibilities arduous or too overwhelming to achieve their prosocial goals; thus, inducing a lower level of health satisfaction [74,76,93,94].

Empirical research supports the relationship between health satisfaction and exit intentions among those who pursue prosocial careers. Poor health satisfaction can be costly in terms of the time and energy needed to perform work-related tasks [95]. Social entrepreneurship requires accessing resources beyond what is currently controlled or possessed, which is mostly rather arduous [96]. Thus, a deficient amount of time and energy for severe challenges mostly induces one’s intention to exit. Therefore, we hypothesize:

**Hypothesis** **2a** **(H2a).** *Social entrepreneurs’ prosocial motivations have a negative effect on their health satisfaction*.

**Hypothesis** **2b** **(H2b).** *Social entrepreneurs’ prosocial motivations have an indirect effect on their exit intentions via health satisfaction*.

### 2.5. The Moderating Role of Entrepreneur’s Gender

According to gender stereotypes, different careers can be perceived as masculine or feminine, conduced to the perceived attractiveness of careers [33,34]. Thus, individuals tend to choose their careers according to their socially recognized gender [97,98]. Prosocial behavior is related to empathy and a sense of social responsibility [99,100]; such values are typically associated with females [33,34]; namely, female entrepreneurs better fit the gender stereotypes of social entrepreneurs [101].

Although people aspire to jobs that are socially acceptable for their genders while avoiding those considered inappropriate [97,98], many engage in occupations that do not conform to gender stereotypes and, thus, may feel stereotype threats [102,103,104]. Previous research claimed that if an individual’s social identity is tagged negatively by gender stereotypes, it could undermine his or her well-being and a sense of belonging [98]. According to Marshall [105], individuals who engage in social entrepreneurship can incur income insecurity over time. For male social entrepreneurs, earning less than a typical commercial entrepreneur may conflict with their gender stereotype as the “breadwinner” [106]; this, in turn, can induce the stereotype threat and amplify the negative effects of prosocial motivation on financial satisfaction [107]. In contrast, females are typically regarded as “caregivers” [106]. Thus, for female entrepreneurs, lower levels of income security due to sustaining social entrepreneurship are unlikely to amplify the negative effects of prosocial motivation on financial satisfaction.

Social entrepreneurship has a higher failure rate than commercial or regular entrepreneurship due to its complexity [108,109]. For male social entrepreneurs, this may conflict with their stereotypical heroic characterizations as income generators [102,110], which in turn can increase goal conflicts and negative emotions induced by prosocial motivation [87,111,112]. This can engender the stereotype threat and, thus, amplify the negative effects of prosocial motivation on health satisfaction [113]. In contrast, as the gender stereotype implies, female entrepreneurs are less likely to be successful in developing profitable firms [108,109], and female social entrepreneurs who fail may have fewer negative emotions resulting from their prosocial motivations [7,53,64]. This, in turn, may ameliorate the negative effects of prosocial motivation on health satisfaction. Therefore, based on the arguments above, we hypothesize:

**Hypothesis** **3a** **(H3a).** *The negative relationship between prosocial motivation and financial satisfaction is stronger for male entrepreneurs than for female entrepreneurs*.

**Hypothesis** **3b** **(H3b).** *The negative relationship between prosocial motivation and health satisfaction is stronger for male entrepreneurs than for female entrepreneurs*.

## 3. Method

### 3.1. Research Framework

**Figure 1:**
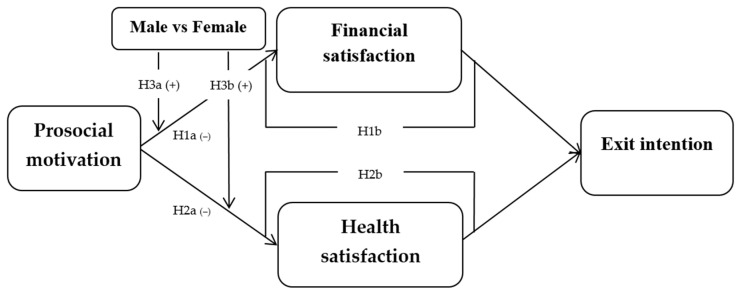
Conceptual model.

Figure 1 shows the conceptual model of this study. First, it posits that prosocial motivation has direct and negative effects on financial satisfaction (H1a) and health satisfaction (H2a). Second, it suggests that prosocial motivation is related to exit intentions via financial satisfaction (H1b) and health satisfaction (H2b). The first path (H1b) predicts that prosocial motivation attenuates financial satisfaction and this, in turn, reinforces exit intention; the second path (H2b) predicts that prosocial motivation diminishes health satisfaction and this, in turn, escalates exit intention. Third, the relationship between prosocial motivation and financial satisfaction is stronger for male entrepreneurs than for female entrepreneurs (H3a), and the relationship between prosocial motivation and health satisfaction is stronger for male entrepreneurs than for female entrepreneurs (H3b).

### 3.2. Sample and Procedure

Via two large-scale colloquiums for entrepreneurs, organized by the All-China Federation of Industry and Commerce (ACFIC) in July and August of 2021, we identified the social entrepreneurs attending the colloquium and surveyed them for this study. The ACFIC is China’s largest semi-official organization, consisting of business owners in diverse industries.

Considering that the respondents of this study were from China and the instruments used in the questionnaire were originally developed in English by prior researchers, we used the approach suggested by Brislin [114] for translating them into Chinese. After the translation was completed, the questionnaire was sent to experts in the field of social enterprise/entrepreneurship for their review. Afterward, a pilot test (on a sample of 100 respondents) was conducted. The Cronbach’s alpha value was over 0.70, indicating the acceptable reliability suggested by Nunnally [115].

Data were gathered during the colloquiums in July (location: Jinan city, Shandong province, China) and August (location: Qingdao city, Shandong province, China), 2021. An invitation (on paper), including a QR code linking to the online questionnaire, was sent to the entrepreneurs (founders or CEOs) participating in the colloquiums.

A total of 450 founders or CEOs accepted our invitation and the response rate exceeded 80%, which is similar to the response rate of prior research [116]. After removing unusable data with missing or problematic values, the sample size was 317 (172 males, 145 females). Table 1 shows an overview of the sample demographics.

For PLS-SEM analyses, Barclay [117] suggested the minimum sample size should be at least 10 times the maximum number of structural paths directed to a construct. The construct with the most paths in our model was the exit intention variable, which had only two paths. Thus, a minimum sample size of 20 was required to validate our model and this study’s sample size (317) was highly sufficient.

Social entrepreneurs were identified with the question below employed by the Global Entrepreneurship Monitor (GEM):

“Are you, alone or with others, currently trying to start or currently owning and managing any kind of activity, organization or initiative that has a particularly social, environmental or community objective? This might include providing services or training to socially deprived or disabled persons, using profits for socially oriented purposes, organizing self-help groups for community action, etc.”

Respondents choosing “no” were identified as conventional or regular entrepreneurs and excluded from this research; while those choosing “yes” were defined as social entrepreneurs and included in this research [118]. This method has also been deployed by prior studies of social entrepreneurs [35,119].

### 3.3. Variables and Measurements

Dependent variable: Exit intention was measured with three items developed by Pollack, Vanepps, and Hayes [52]. The items were rated on a Likert 7-point scale ranging from 1 = strongly disagree to 7 = strongly agree. The Cronbach’s alpha for this scale was 0.927.

Independent variable: Prosocial motivation was measured with four items developed by Adam and Grant [27,120]. The items were rated on a 7-point scale ranging from 1 (strongly disagree) to 7 (strongly agree). The Cronbach’s alpha for this scale was 0.850.

Mediating variable: Financial satisfaction was measured using the one-item measure developed by Fors, Johansson Sevä, and Gärling [70]. Participants indicated their satisfaction with their “private financial situation” on a 7-point scale ranging from 1 = extremely dissatisfied to 7 = extremely satisfied.

Mediating variable: Health satisfaction was measured by requesting the respondents to report the current state of their health [86,121] on a 7-point scale ranging from 1 = completely dissatisfied to 7 = completely satisfied.

Moderating variable: Gender. Male respondents were coded as “1” and female respondents were coded as “2”.

A summary of the operational definitions is presented in Table 2. Moreover, the English version of the items has been appended (Appendix A).

## 4. Data Analysis

To test our hypotheses, we employed consistent bootstrapped partial least square structural equation modeling (PLS-SEM) using the software SmartPLS (Version 3.3.3) [122]. Research suggests that PLS-SEM is increasingly being deployed in entrepreneurship research [123], and it is considered suitable for analyzing models with complex paths [123]. It is not limited by stringent assumptions (e.g., the multivariate normality) and sample size requirements [123].

Specifically, there were two reasons to use PLS-SEM for data analyses: first, PLS-SEM has been found to be effective in testing complex models, allowing simultaneous estimations of multiple causal relationships between variables [124], such as the ones in this study. Second, PLS-SEM is suitable for the exploratory analyses [122], such as the one in this study.

The analysis was conducted through two stages [117]. (1) The analysis of the outer model tested the reliability and validity of all latent construct measurements. (2) The analysis of the inner model assessed the relationships among the latent constructs for hypothesis testing. This sequence was to ensure that the measurement scales were valid and reliable.

### 4.1. Outer Model Analysis

The outer model’s validity was evaluated by testing the reliability of each construct, the internal consistency of measures, and the convergent and discriminant validities of each construct.

First, the reliability of constructs was evaluated by examining the factor loadings of each indicator. As Table 3 shows, all factor loadings (range: 0.769 to 0.962) reached the threshold value suggested by Hair et al. [125] of 0.70, implying adequate reliability.

Second, the internal consistency of the measures was examined by computing composite reliability (CR) values (Table 3). The composite reliability values were 0.899 (prosocial motivation) and 0.954 (exit intention), above the acceptable threshold value (0.80), as suggested by Fornell and Larcker [126].

Third, convergent validity was examined by computing the average variance extracted (AVE) values. Table 3 shows the AVEs were 0.690 (prosocial motivation) and 0.873 (exit intention) above the acceptable threshold (0.50) [126], indicating sufficient convergent validity.

Fourth, discriminant validity was tested by comparing the cross-loadings and factor loadings for each indicator (See Table 4), and the heterotrait-monotrait (HTMT) ratio of correlations (See Table 5). As shown in Table 4, the factor loading of each scale item for its assigned latent construct was higher than its loading on any other construct [122], suggesting good discriminant validity. Moreover, as shown in Table 5, the HTMT ratios of the average correlations of the indicators across constructs were all below the threshold (0.90) [127], indicating that each construct was empirically distinct from other constructs in the model and the discriminant validity was sufficient.

### 4.2. Inner Model Analysis

**Figure 2:**
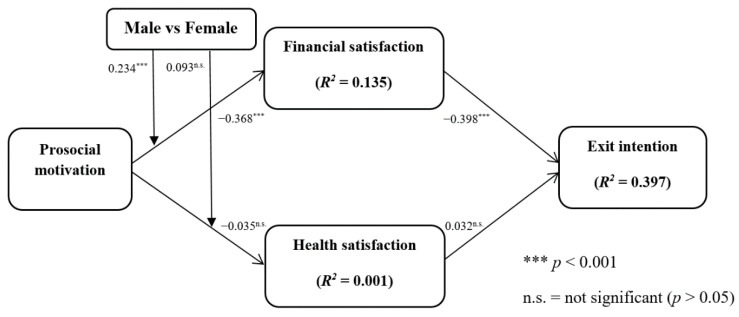
Path coefficients and *R*^2^ of the inner model.

The inner model was assessed by computing *R*^2^, effect size (*f*^2^), *Q*^2^, and path coefficients. The *R*^2^ value of endogenous constructs is viewed as the primary criteria for assessing the quality of structural models [128]. We chose to follow the guidelines suggested by Chin [129]; the endogenous latent variables are considered reliable if their *R*^2^ values are greater than 0.10 [130]. Meanwhile, for exploratory studies in social sciences, the *R*^2^ value lower than 0.10 is also accepted [130]. Thus, as shown in Figure 2, the *R*^2^ values indicate the significant explanatory power of the model.

Cohen’s *f*^2^ was used to evaluate the contribution of an exogenous variable in multiple regression models. Cohen’s guidelines suggest the following criteria for evaluating *f*^2^ values: weak = 0.02; medium = 0.15, and large = 0.35 [131]. The *f*^2^ value for H1a: PM → FS was 0.218, indicating a medium-level contribution of prosocial motivation to predicting financial satisfaction. In contrast, for H2a: PM → HS, *f*^2^ was 0.002, indicating a negligible contribution of prosocial motivation to predicting health satisfaction.

As part of checking the predictive relevance, the *Q*^2^ values were also computed. The *Q*^2^ values for financial satisfaction, health satisfaction, and exit intention were 0.126, 0.013, and 0.341, respectively. Given that all of them were greater than zero, the explanatory constructs had adequate predictive relevance for their indicators [132].

Goodness of Fit (GoF) (0 < GoF <1) is another indicator of a PLS-SEM model’s quality [133]. The GOF is calculated as:

$$\mathrm{GoF}=\sqrt{\bar{communality}\times \bar{R^{2}}}=0.79$$

The GoF values of 0.10, 0.25, and 0.36 are defined as small, medium, and large effect sizes, respectively [134]. The GoF value for the proposed model was 0.79, indicating a large effect size. Based on the above findings, it can be concluded that the proposed model has a good overall fit.

Next, we examined the structural relationships in the proposed model. Figure 2 reports the results of the algorithm and bootstrapping tests (based on 5000 samples), including the path coefficients (β), *t*-values, and retention or rejection of each hypothesis. Purvis et al. [135] suggested that bootstrapping is an effective procedure to evaluate the significance of each path coefficient. Figure 2 presents the bootstrapping validation outcomes. H1a (predicting prosocial motivation and negatively related to financial satisfaction) was supported (PM → FS: β = −0.368, *t*-value = 8.091, *p* < 0.001). H2a (predicting prosocial motivation and negatively related to health satisfaction) was not supported (PM → HS: β = −0.035, *t*-value = 0.632).

### 4.3. Mediation Effects

The Sobel test and variance accounted for (VAF) index were employed [136] to examine the mediation hypotheses (H1b and H2b). Per Sobel’s test (See Table 6) [136], the mediation by financial satisfaction was significant (absolute Z value = 4.173, *p* < 0.01); whereas the mediation by health satisfaction was not significant (absolute Z value = 0.363, *p* > 0.05).

The method of variance accounted for (VAF) suggested by Hair Jr., Hult, Ringle, and Sarstedt [122] was used to determine the strength of the indirect effects (i.e., mediation effect) in relation to the total effect (i.e., direct effect plus indirect effect). The recommended VAF cutoff values for determining mediation effects are as follows: full mediation >80%, partial mediation ≤80%, and no mediation <20% [122]. Table 6 shows that financial satisfaction was a partial mediator in the prosocial motivation–exit intention relation, supporting hypothesis H1b. However, hypothesis H2b, concerning the mediation effects of health satisfaction, was not supported since the VAF was less than 20%.

### 4.4. Moderation Effects

The multiple group analysis procedure (PLS-MGA) in SmartPLS (Version 3.3.3) was used to examine if the path coefficients [137] for males and females (1 = male, and 2 = female) differed significantly. PLS-MGA was conducted with a bootstrapped sample of 5000 cases. This analysis allowed us to see which path was distinct, how different the paths were, and whether there was a difference in the path direction. The results are presented in Table 7.

The results of the PLS-MGA indicate that the path between prosocial motivation and financial satisfaction was significantly stronger for males than for females, with a coefficient difference of 0.234 (*p* = 0.003). Therefore, H3a was supported. However, there was no statistically significant difference between males and females in the path coefficients between prosocial motivation and health satisfaction. Accordingly, H3b was not supported.

## 5. Discussion

This study reflects our attempt to respond to the criticism that social entrepreneurship research has been constricted and to open the “black box” regarding the relationship between prosocial motivation and exit intentions [6,9]. Scholars have noted that “[o]ur desire—our need—to open up the black box is not just a matter of scholarly curiosity; it is essential for ultimately improving the insights we can provide…” [138]. Largely, this research represents a substantive step in this direction.

### 5.1. Theoretical Implications

Although scholars who study social entrepreneurship have already highlighted the effects of prosocial motivation on entrepreneurs’ exit intentions [6,9], they have largely overlooked the mechanism behind this relationship. Our study adopted the theoretical perspective, the hierarchical approach to personality [21,22], to explore such a mechanism between entrepreneurs’ personality traits and entrepreneurial outcomes. Our findings indicate that prosocial motivation (as a personality trait) can be linked to the entrepreneurial outcome exit intention through entrepreneurs’ financial satisfaction. Thus, our findings advance extant scholarships, especially concerning the relationship between personality traits and entrepreneurial outcomes [17].

Specifically, we found that financial satisfaction mediated the nexus between prosocial motivation and exit intentions. These findings are well in line with the literature [50,75,76]. A simple but plausible explanation for these results is that social entrepreneurs need adequate financial support to handle a wide range of financial challenges [50,61], easily leading to a lower level of financial satisfaction [76]. If financial satisfaction runs low for social entrepreneurs, it could erode their confidence (in their own competence), encouraging them to regret the career paths they have chosen [82,83], thereby engendering their exit intentions.

Furthermore, we found that the negative impact of prosocial motivation on financial satisfaction was stronger for male entrepreneurs than for female social entrepreneurs. This suggests that gender stereotypes about occupational choice can enhance the negative impact of being a social entrepreneur (on financial satisfaction), possibly leading to stronger intentions to exit. These results respond to previous researchers’ calls as to why social enterprises are more likely to be started by men than by women, but the gender gap throughout the entrepreneurial life cycle is not large anymore [30]. Our findings further show the potential gender stereotype threat and relevant issues in the context of social entrepreneurship.

Contrary to our prediction, we found that health satisfaction did not mediate the relationship between prosocial motivation and exit intention. This is possibly due to the age of the sampled entrepreneurs. Although satisfaction with one’s health is normally based on one’s actual health status, the strength of this relationship might not be the same across the age range. Prior studies claimed that health satisfaction trajectories are relatively flat throughout the lifespan before age 50 and then decrease sharply afterward until the end of one’s lifespan [139,140]. This is because people over 50 are particularly intolerant of the early signs of aging [140]. In our study, over 65% of the respondents were below 45 years old; therefore, the health satisfaction of these entrepreneurs could be inflated.

### 5.2. Practical Implications

According to our findings, prosocial motivation, the typical personality trait of social entrepreneurs, can cause exit intention via life-related wellbeing, such as financial satisfaction. Thus, entrepreneurship educators may need to be aware of the mechanisms, given the high possible failure rate of social entrepreneurs in achieving prosocial goals. Moreover, only focusing on successful case studies for training programs on entrepreneurship can be problematic and misleading. Given our findings that financial satisfaction was a significant mediator, it is necessary to develop the social entrepreneurs’ capabilities to acquire financial and institutional support and to encourage them to develop budgeting policies to achieve prosocial goals. Furthermore, given our finding that male social entrepreneurs may have a lower level of life satisfaction compared to female entrepreneurs, relevant government agencies should provide greater support, including relevant policies, facilities, training programs, and consultation availabilities to promote gender role equality and life satisfaction.

## 6. Limitations and Future Research Directions

This study is not without limitations. First, our analyses should be replicated with different samples from various countries. The distinctive characteristics of China’s society, culture, and lifestyle may help explain the findings of this study. As different economic, cultural, and institutional business environments can affect socially-oriented entrepreneurial activities differently [119,141,142,143,144], further research could involve other economic, cultural, and institutional contexts to test the generalizability of our findings.

Second, besides the variables included in this research, other social, biological, occupational, and professional factors may influence the path from prosocial motivation to exit intention. Therefore, additional levels of analysis would further help explicate the individual vs. contextual influences.

Third, future studies involving the potential dimensions of wellbeing or satisfaction as the mediators, and unveiling how they function uniformly or differentially, can help to further our understanding of the nuances in these relationships.

Fourth, in future research, human capital features, such as education, experience, and skills, need to be included as the control variables to further our findings.

## 7. Conclusions

We found that prosocial motivation negatively influenced the financial satisfaction of social entrepreneurs, which in turn was associated with an increase in their exit intentions. This relationship was significantly stronger for male entrepreneurs than for female entrepreneurs.

## Figures and Tables

**Table 1:** Sample demographics.

Characteristics	Frequency	Percent (%)
Age		
18–25	5	1.6%
26–35	110	34.7%
36–45	71	22.4%
46–55	131	41.3%
Gender		
Male	172	54.3%
Female	145	45.7%
Marital status		
Married	217	68.5%
Non-married	100	31.5%
Educational Level		
Junior high school	0	0%
High school or equal	2	0.6%
Junior college	61	19.2%
Bachelor’s degree	139	43.9%
Postgraduate or above	115	36.3%

**Table 2:** Operational definitions.

Construct	Definition	Source
Exit intention	An entrepreneur’s desire or goal, at some point in the future, to leave his or her venture.	[20,52]
Prosocial motivation	The desire to help others or expend effort out of concern for others.	[7]
Financial satisfaction	A cognitive evaluation of one’s present financial situation.	[70]
Health satisfaction	A cognitive judgment of individuals about the quality of their overall mental and physical fitness.	[85,86]

**Table 3:** Reliability and AVE of the measurement model (outer model).

Construct	Indicators	Factor Loading	Composite Reliability	AVE
PM	PM 1	0.868	0.899	0.690
	PM 2	0.835	-	-
	PM 3	0.769	-	-
	PM 4	0.851	-	-
EI	EI 1	0.903	0.954	0.873
	EI 2	0.962	-	-
	EI 3	0.936	-	-

Note 1: PM = prosocial motivation; EI = exit intention. Note 2: Financial satisfaction is a single-item construct. Note 3: health satisfaction is a single-item construct.

**Table 4:** Discriminant validity—factor loadings and cross-loadings.

	EI	FS	HS	PM
EI1	0.903	−0.504	−0.031	0.445
EI2	0.962	−0.500	−0.012	0.492
EI3	0.936	−0.433	−0.020	0.501
FS1	−0.527	1	0.169	−0.368
HS1	−0.023	0.169	1	0.035
PM1	0.465	−0.291	0.091	0.868
PM2	0.365	−0.246	0.012	0.835
PM3	0.393	−0.340	−0.012	0.769
PM4	0.469	−0.333	0.027	0.841

Note 1: PM = prosocial motivation; FS = financial satisfaction; HS = health satisfaction; EI = exit intention. Note 2: the grey cells are the factor loadings of scale items for each construct.

**Table 5:** Discriminant validity—HTMT.

Factors	EI	FS	HS	PM
EI	-	-	-	-
FS	0.546	-	-	-
HS	0.025	0.169	-	-
PM	0.575	0.396	0.049	-

Note 1: PM = prosocial motivation; FS = financial satisfaction; HS = health satisfaction; EI = exit intention.

**Table 6:** Test of mediation effect.

	Original Sample (O)	Standard Error (STERR)	*t* (|O/STERR|)	
PM → FS	−0.368	0.062	5.903	-
FS → EI	−0.398	0.067	5.900	-
PM → HS	0.035	0.074	0.467	-
HS → EI	0.032	0.056	0.577	-
PM → EI	0.366	0.051	7.232	-
	PM → FS → EI	-	PM → HS → EI	Total indirect effect
Indirect effect	0.146	-	0.001	0.515
Sobel Z Test	4.173	-	0.363	-
VAF	0.285	-	0.002	0.287
Supported	YES	-	NO	

Note 1: PM = prosocial motivation; JS = job satisfaction; WB = work burnout; WA = work anxiety; EI = exit intention. Note 2: number of bootstrap samples = 5000.

**Table 7:** Results of the multi-group analysis.

Path	Pooled		Males (M)			Females (F)			M vs. F	Supported
	*n* = 317		*n* = 145			*n* = 172				
	β	CI	β	CI	*f* ^2^	β	CI	*f* ^2^	*p*-Value	
PM → FS	−0.368	(0.092, 0.202)	−0.437	(−0.555, −0.310)	0.236	−0.203	(−0.340, −0.041)	0.043	0.003	YES
PM → HS	−0.035	(−0.004, 0.009)	0.007	(−0.152, 0.190)	0.010	0.100	(−0.057, 0.241)	0.007	0.413	NO

Note 1: PM = prosocial motivation; FS = financial satisfaction; HS = health satisfaction; EI = exit intention. Note 2: β = path coefficient; CI = 95% Confidence interval. Note 3: *f*^2^ = size effect: 0.02 < *f*^2^ < 0.15 (small effect size); 0.15 < *f*^2^ < 0.35 (medium effect size); *f*^2^ > 0.35 (large effect size).

## Data Availability

The data will be made available upon request from the first author.

## Appendix A

**Table A1:** The Questionnaire.

Construct	Items	Variables	References
Prosocial Motivation (PM)	I care about benefiting others through my work I want to have a positive impact on others Because I want to have a positive impact on others It is important to me to do good for others through my work	PM1–PM4	[27]
Financial Satisfaction (FS)	How comfortable and well-off are you financially?	FS1	[70,120]
Health Satisfaction (HS)	How would you rate your level of satisfaction with your own health?	HS1	[86,121]
Exit Intention (EI)	Participants rated the extent to which they would, in the next year. Avoid entrepreneurial positions Feel anxious about entrepreneurial positions Feel less excited about entrepreneurial positions	EI1–EI3	[52]
